# Deficiency in the metabolite receptor SUCNR1 (GPR91) leads to outer retinal lesions

**DOI:** 10.18632/aging.100563

**Published:** 2013-06-17

**Authors:** Sandra Favret, Francois Binet, Eric Lapalme, Dominique Leboeuf, Jose Carbadillo, Tina Rubic, Emilie Picard, Gaelle Mawambo, Nicolas Tetreault, Jean-Sebastien Joyal, Sylvain Chemtob, Florian Sennlaub, John Paul SanGiovanni, Martin Guimond, Przemyslaw Sapieha

**Affiliations:** ^1^ Department of Ophthalmology, Hopital Maisonneuve-Rosemont Research Centre, University of Montreal, Montreal, Quebec, H1T 2M4, Canada; ^2^ Department of Biochemistry, Hopital Maisonneuve-Rosemont Research Centre, University of Montreal, Montreal, Quebec, H1T 2M4, Canada; ^3^ Department of Immunology, Hopital Maisonneuve-Rosemont Research Centre, University of Montreal, Montreal, Quebec, H1T 2M4, Canada; ^4^ Novartis Institutes for Biomedical Research, Basel CH-4002, Switzerland; ^5^ Université Pierre et Marie Curie et Université Descartes, Paris, France; 2 INSERM, CRC UMRS872 team 17, Paris, France; ^6^ INSERM, U 968, Paris, F-75012, France CNRS, UMR_7210 & UPMC Univ Paris 06, UMR_S 968, Institut de la Vision, Paris, F-75012, France; ^7^ AP-HP, Hôtel Dieu, Service d'Ophtalmologie, Centre de Recherche ophtalmologique, 75006 Paris; ^8^ Clinical Trials Branch and Clinical Research, National Eye Institute, Bethesda, MD 20892

**Keywords:** AMD, GPR91, geographic atrophy, microglia, succinate, metabolite receptor.

## Abstract

Age-related macular degeneration (AMD) is a prominent cause of blindness in the Western world. To date, its molecular pathogenesis as well as the sequence of events leading to retinal degeneration remain largely ill-defined. While the invasion of choroidal neovasculature in the retina is the primary mechanism that precipitates loss of sight, an earlier dry form may accompany it. Here we provide the first evidence for the protective role of the Retinal Pigment Epithelium (RPE)-resident metabolite receptor, succinate receptor 1 (SUCNR1; G-Protein coupled Receptor-91 (GPR91), in preventing dry AMD-like lesions of the outer retina. Genetic analysis of 925 patients with geographic atrophy and 1199 AMD-free peers revealed an increased risk of developing geographic atrophy associated with intronic variants in the *SUCNR1* gene. In mice, outer retinal expression of SUCNR1 is observed in the RPE as well as microglial cells and decreases progressively with age. Accordingly, *Sucnr1−/−* mice show signs of premature sub-retinal dystrophy with accumulation of oxidized-LDL, abnormal thickening of Bruch's membrane and a buildup of subretinal microglia. The accumulation of microglia in *Sucnr1*-deficient mice is likely triggered by the inefficient clearance of oxidized lipids by the RPE as bone marrow transfer of wild-type microglia into *Sucnr1−/−* mice did not salvage the patho-phenotype and systemic lipolysis was equivalent between wild-type and control mice. Our findings suggest that deficiency in SUCNR1 is a possible contributing factor to the pathogenesis of dry AMD and thus broaden our understanding of this clinically unmet need.

## INTRODUCTION

Age-related macular degeneration is the leading cause of blindness in developed countries, affecting over 10 million individuals in North America itself and a proportional number in Europe [1-3]. The aging of “baby boomers” will lead to a doubling of the population of 65 years of age or older by 2031 and thus significantly increase the number of affected individuals. AMD, as its name suggests, is diagnosed secondary to macular lesions [4] and is divided into two distinct, yet associated, forms based on their phenotypic manifestation; a dry atrophic form and a wet exudative form [5-7].

The dry form of AMD is characterized by drusen which are stereotypic extracellular deposits between the RPE basal lamina and the inner collagenous layer of Bruch's membrane[7]. Drusen can also result from membranous debris shed from the basal surface of the RPE and eventually lead, over time, tolarge areas of retinal degeneration called geographic atrophy (GA) [5, 7]. Of note, it is now believed that drusenoid deposits (i.e. reticular pseudodrusen) can also be found on the apical side of RPE and contribute to AMD progression [8].

The exudative (or wet/neovascular) form of AMD is the most widely associated with central vision impairment and legal blindness. Here, unchecked vasoproliferation of choroidal vessels specifically in the macular area, directly leads to photoreceptor compromise and vision loss. Although considerable effort has been invested in deciphering the molecular and cellular mechanisms governing neovascular AMD,substantially less is known on the pathogenesis of atrophic AMD. Consequently, while effective anti-angiogenic treatment modalities are presently available for wet AMD [6], there is currently a void of effective therapeutic options to counter atrophic AMD. Designing an efficient therapeutic strategy would answer a clinically unmet need.

The precise sequence of cellular events leading to non-neovascular AMD and GA remains ill-defined. In a healthy retina, outer-segment discs of photoreceptors are phagocytized by the apical surface of RPE and degraded at very high rates [9]; estimated at upward of 30,000 per day in rats [10]. Debris is subsequently cleared by choroidal circulation. Photoreceptors preserve a constant length through the generation of new outer segments and by discharging spent discs. Inefficient phagocytosis of used outer-segments by RPE leads to sub-RPE accumulation of oxidized lipids as does deficiency in key lipid scavenger receptors such as CD36 [11, 12]. A number of plasma proteins have also been reported to accumulate in drusen including vitronectin [13], clusterin [14] and serum amyloid P [15], suggesting a systemic contribution to their formation. Subsequently, a sequential appearance of dendritic cell processes, class II antigens, components of the complement system, and coagulation factors has been reported [16]. Consistent with a cellular immune response associated with early sub-RPE deposit, mice genetically deficient for either CC chemokine receptor 2 (CCR2) or its cognate ligand CC chemokine ligand 2-(CCL-2) show premature accumulation of lipofuscin and drusen formation [17]. Moreover, resident microglia amass in the subretinal space of *CX3CR1-*deficient mice at sites of drusen formation and retinal degeneration [18].

Given the compromised myeloid cell function and aberrant lipid processing associated with the progression of atrophic AMD [18, 19], we sought to investigate the role of the metabolite receptor SUCNR1 (GPR91) in its pathogenesis. SUCNR1 is the cognate G-protein coupled receptor for succinate [20] although other ligands likely exist. SUCNR1 has been characterized as an immunomodulatory receptor that induces a migratory response in dendritic cells and acts in the production of pro-inflammatory cytokines [21]. Moreover, SUCNR1 is highly expressed in adipocytes and white adipose tissue (WAT) and addition of succinate to WAT has been shown to inhibit lipolysis in a SUCNR1-dependent manner in isolated WAT [22]. Moreover, we have previously demonstrated that SUCNR1 modulates the expression of pro-angiogenic factors in a HIF-1α-independent manner and its deletion protects against pathological pre-retinal neo-vascularization [23].

Here we demonstrate for the first time, the contribution of a metabolite-sensing receptor in mediating AMD-like lesions of the sub-retinal space. In doing so, we propose a sequence of events where deficient lipid processing by *Sucnr1−/−* RPE instigates subretinal accumulation of microglia. We provide evidence that human DNA sequence variants in *SUCNR1* are associated with atrophic AMD and demonstrate that *Sucnr1−/−* mice readily accumulate subretinal oxLDL, display thickening of Bruch's membrane (BM), and collect abnormally elevated levels of sub-retinal microglial cells. In addition, *Sucnr1−/−* microglia showed overall compromised motility, which likely contributes to their trapping. Hence, although likely a minor contributor to the overall disease process, deficiency in SUCNR1 may be a predisposing factor for atrophic AMD.

## RESULTS

### DNA Sequence Variants in *SUCNR1* are Associated with Atrophic AMD in Humans

To evaluate the involvement of *SUCNR1* (*GPR91*) in dry AMD, we applied age-, sex-, and smoking-adjusted logistic regression models to analyze sequence variation in *SUCNR1* for association with atrophic AMD. Our samples were collected from four independent cohorts participating in large-scale genotyping projects on the molecular genetics of AMD. We analyzed 925 people with geographic atrophy (GA) and 1199 of their elderly peers who were both AMD-free and ≥ 65 years-of-age, computing combined estimates of association across cohorts with meta-regression and deriving exact *P*-values with max(T) permutation on 10000 iterations. GA-*SUCNR1* relationships emerged for two of the three common DNA sequence variants we tested (rs13315275, *P*_exact_=0.005; rs9811297, *P*_exact_=0.031). The finding for rs13315275 persisted after adjustment for family-wise error (*P*_exact_=0.011) (Table 1). The minor allele frequencies of *SUCNR1* sequence variants were tested for association with atrophic age-related macular degeneration and presented in Table 2.

**Table 1:** Succinate receptor 1 (GPR91) DNA variants tested for association with atrophic age-related macular degeneration

OR							Exact Test		
Variant	Alleles	Cohort	Cohort	Cohort	Cohort	Meta	*P*meta	*P*point	*P*adj
		1	2	3	4				
rs981129	A|G	1.14	1.07	1.47	1.61	1.23	0.007	0.005	0.011
rs9811297	A|G	--	1.63	1.25	1.61	1.51	0.026	0.031	0.093
rs1445358	A|G	--	1.04	0.77	1.56	1.08	0.660	0.690	0.967

Note: Odds ratios (OR) are from age-, sex-, and smoking-adjusted logistic regression analyses examining the likelihood of having geographic atrophy (additive model). Minor alleles are listed first in the ‘Allele’ column. All people in the AMD-free (control) group were 65-years-of-age or older. Cohort 1 (Age-Related Eye Disease Study, 391 with geographic atrophy (GA), 189 AMD-free). Cohort 2 (University of Michigan Clinic, 329 with GA, 508 AMD-free). Cohort 3 (University of Pennsylvania Clinic, 110 with GA, 194 AMD-free). Cohort 4 (Mayo Clinic, 95 with GA, 308 AMD-free). Meta = results from meta-analysis. *P*_point_ = point-wise exact *P*-value. *P*_adj_ = exact *P-values* adjusted for family-wise error. All *P-values* are 2-sided. Minor allele frequencies are presented in Table 2. -- = not tested.

**Table 2:** Minor allele frequencies of succinate receptor 1 (GPR91) sequence variants tested for association with atrophic age-related macular degeneration

		Cohort							
Variant	Alleles	1		2		3		4	
		GA	No AMD	GA	No AMD	GA	No AMD	GA	No AMD
rs13315275	A|G	0.44	0.40	0.42	0.41	0.49	0.39	0.51	0.40
rs9811297	A|G	--	--	0.14	0.14	0.18	0.14	0.21	0.13
rs1445358	A|G	--	--	0.19	0.16	0.19	0.16	0.25	0.17

GA = geographic atrophy

In addition, we used the CEU cohort (people of similar ancestry to our analytic samples) from the 1000 Genomes Project to identify sequence variants in nearly complete linkage disequilibrium (r^2^ > 0.90) with our GA-associated SNPs. rs13315275 is co-inherited with rs7638353, a variant showing a moderate signal for a DNase hypersensitivity cluster in human RPE cells (HRPEpiC, ScienCell) analyzed with DNase-seq in the ENCODE Project. Promoter regions tend to be DNase-sensitive. rs9811297 is in nearly complete linkage disequilibrium with at least two variants resident in the 5' untranslated region (UTR) of SUCNR1 (rs1402012 and rs2120979 The findings demonstrate that GA-associated SNPs first identified in our cohorts may be co-inherited with non-coding variants in SUCNR1 that are influenced by regulatory elements. Together, these human data suggest that perturbation in the *SUNCR1* gene may be linked to the progression of atrophic AMD. This is in agreement with current AMD-genome wide analyses that suggest involvement of loci near genes implicated in angiogenesis (VEGFA, TGFBR1) and lipid metabolism (ApoE, CETP) [24]; both processes are modulated by SUCNR1.

### SUCNR1 is Expressed in Retina and Microglia and Diminishes with Age

**Figure 1:**
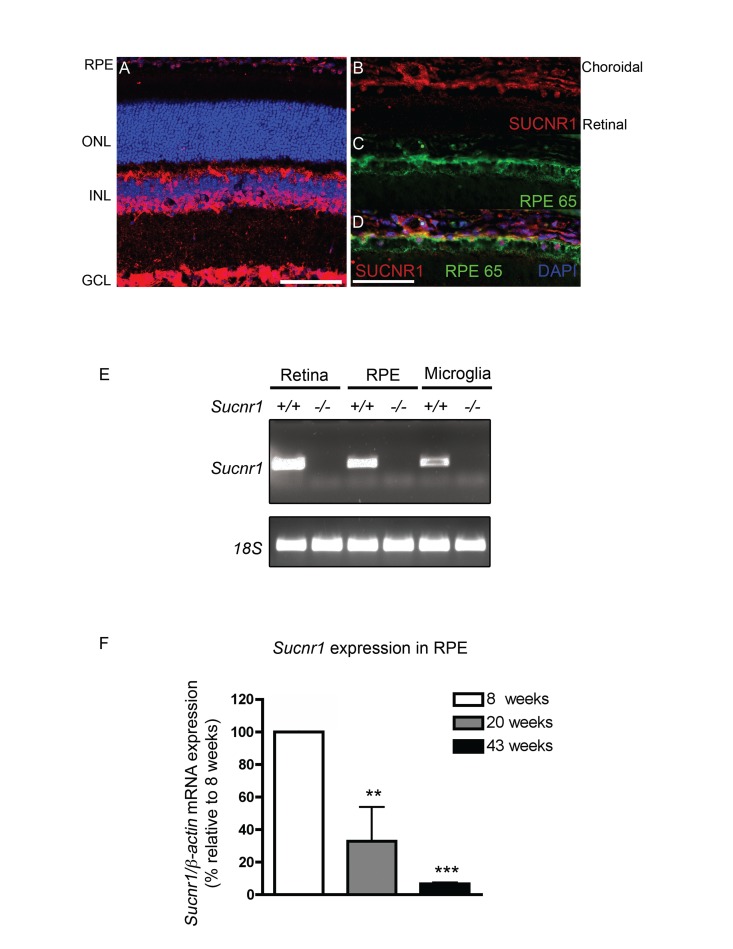
SUCNR1 is expressed in RPE and microglia and diminishes with age (**A**) Immunohistochemistry on sagittal retinal cryosections reveals expression of SUCNR1 in the GCL, INL and RPE. (**B-D**) Confocal imaging corroborates expression of SUCNR1 (red) in the RPE as confirmed by co-localization with the RPE marker RPE65 (green). Images are representative of 3-4 experiments. (**E**) Expression profile of *Sucnr1* mRNA shows transcripts in RPE and CNS microglia from wild-type mice while an absence of transcripts is noted in samples from *Sucnr1−/−* mice. (**F**) Quantitative PCR on RPE isolated from 8, 20 or 43 week old wild-type mice shows a steady decrease of *Sucnr1* levels with age (n=4-6). Values are expressed as percentage of controls ± S.E.M., normalized to β-actin standards. ONL: outer nuclear layer, INL: inner nuclear layer, GCL: ganglion cell layer and RPE: retinal pigment epithelium. Scale bars (**A-D**): 100μm.

To establish a prospective patho-mechanism by which deficiency in *Sucnr1* could contribute to AMD-like lesions of the outer retina, we first investigated the retinal localization and expression patterns of this GPCR. Immunohistochemical analysis on sagittal retinal sections revealed notable expression of SUCNR1 in the retinal ganglion cell layer (GCL), cells of the inner nuclear layer (INL) and RPE (Figure 1A) as previously described [23, 25]). In line with a role in contributing to outer-retinal homeostasis, SUCNR1 was expressed in the RPE (co-localization with RPE65) (Figure 1B-D) confirming a previous report [25]. Beyond expression in the retina proper, presence of SUCNR1 was also noted in microglial cells (isolated from the brain) that have been previously implicated in the pathogenesis of AMD [18] (Figure 1E). Specificity of expression was confirmed using analogous tissue from *Sucnr1−/−* mice.

Given that AMD-related lesions by definition appear with age, we next explored the expression dynamics of *Sucnr1* mRNA in the outer retina of mice at various ages. Expression of *Sucnr1* in isolated RPE decreased persistently with time. Levels of RPE-derived *Sucnr1* mRNA dropped by 3-fold from 8 to 20 weeks of age (P=0.0265) and were 15-fold lower at 43 weeks when compared to 8 weeks (P=0.0010) (Figure 1F). Taken together, these data suggest that the expression of SUCNR1 is consistent with a potential role in atrophic AMD.

### SUCNR1 Deficiency Leads to Subretinal Lesions and Accumulation of oxLDL

**Figure 2:**
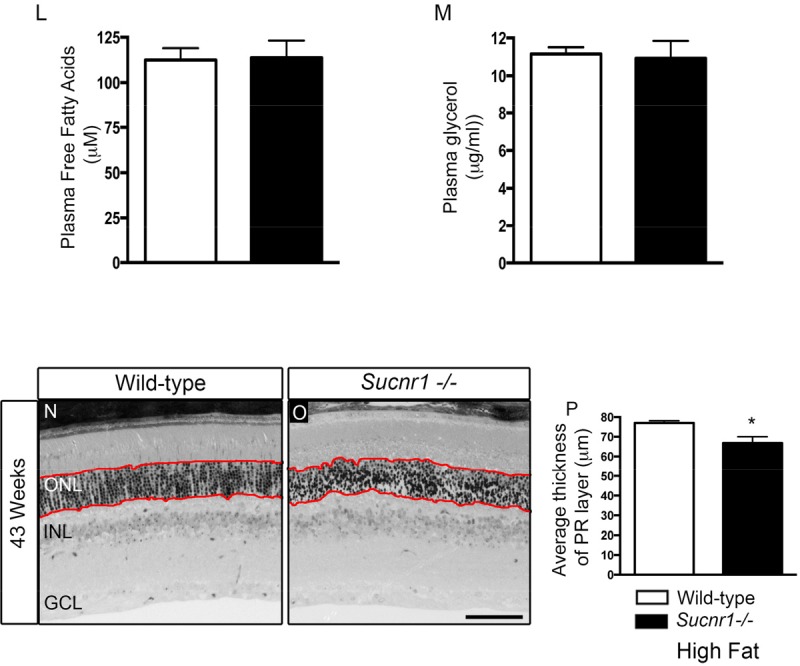
Deficiency in SUCNR1 leads to outer retinal lesions Transmission electron microscopy of RPE/sub-retina the in wild-type (**A**) and *Sucnr1−/−* (**B**) at 20 weeks of age reveals regional disruption of BM and presence nodular debris (asterix). By 43 weeks of age, lipofuscin granules (black arrows) accumulate in the sub-retina of *Sucnr1−/−* mice (**D**) while minimal lipofuscin is detected in wild-type mice. Confocal microscopy on retinal cross sections demonstrates accumulation of sub-retinal deposits of oxLDL in Sucnr1−/− mice (**F,H,J**) while wild-type controls remained ox-LDL free (**E,G,I**). Images are representative of 3 distinct experiments. (**K**) Quantitative PCR reveals lower levels of scavenger receptor CD36 in isolated RPE extracts in *Sucnr1−/−* mice when compared to wild-type controls (n=5-10). Values are expressed as percentage of controls ? S.E.M, normalized to ?-actin standards. *P<0,05. Similar levels of plasma FFA (**L**) and glycerol (**M**) were noted in both wild-type and *Sucnr1−/−* mice suggesting that systemic lipolysis was not affected by SUCNR1. (**N-P**) Toluidine blue-stained epoxy retinal semi-thin sections show mild degeneration of photoreceptors (20 points of analysis per retina; n=3-4 mice) *P<0,05. Scale bar (**A-D**): 2μm; (**E-J**): 100μm; (**N,O**): 75μm.

In light of genetic evidence and an expression pattern consistent with a role in mediating features of dry AMD, we sought to determine if *Suncr1*-deficient mice displayed signs of degeneration of the outer retina. Inefficient degradation of photoreceptor outer segments by the RPE results in the formation of heterogeneous sub-RPE cellular waste products including lipofuscins which accumulate in RPE with age and may contribute to AMD pathogenesis [13, 26]. Detailed morphological evaluation by electron microscopy (EM) of *Sucnr1−/−* retinas revealed perturbation in the structure of the outer retina (Figure 2). Lesions in the outer retina were noted as early as 20 weeks of life and included nodular debris (asterix) in the BM (Figure 2A,B). By 43 weeks of life, *Sucnr1−/−* mice showed increased accumulation of lipofuscin (black arrows) while they were minimally detected in wild-type controls (Figure 2C,D).

An important risk factor for developing AMD is hypercholesterolemia. In this respect, human ApoE4 expressing mice show a profound degeneration of the RPE layer after a high-fat, cholesterol-rich diet[27]. We therefore fed mice high fat diets (36% fat; fat calories 60%) from the 8^th^ week of life to accelerate and exacerbate sub-retinal lipid accumulation. By 43 weeks of life, oxLDL deposits were found in the sub-RPE space of *Sucnr1−/−* mice suggesting deficits in lipid elimination (Figure 2E-J). The build-up of oxidized lipids in the outer retina coincided with significantly lower levels in the RPE of the principal scavenger receptor for oxidized lipids CD36, [11, 12]. CD36 transcripts in RPE isolated from *Sucnr1−/−* mice decreased by ~40% under control diets (P=0.0235) and ~60% in mice fed high fat diets (P=0.0299) (Figure 2K).

Given that SUCNR1 was speculated to affect lipolysis [22], we sought to determine if systemic levels of free fatty acids (FFA) and glycerol varied in our paradigm and could account for increased outer retinal lipid deposition. Mice were fasted for 12 hours during the light cycle, and received an intra-peritoneal injection of 3 mg/g of glucose at the beginning of the dark cycle. Plasma levels of FFA and glycerol were measured 30 minutes after glucose injection and did not vary between wild-type and *Sucnr1*-deficient mice (Figure 2L,M). Hence, variations in lipolysis efficiency could not account for the lipid accumulation observed in the outer retinas of Sucnr1−/− mice. Importantly, while the SUCNR1−/− mice were found to have the *rd8* mutation of the *Crb1* gene [28], the lesions observed in the outer retina translated only into a mild (~13% decrease; P=0.0196), loss of photoreceptor cells in *Sucnr1−/−* mice and only in 43 week-old high fat-fed mice as determined by histological analysis on semi-thin sections (measurements taken within red lines; (Figure 2N-P).

### Deficiency in Sucnr1 Leads to Bruch's Membrane Thickening

The BM is comprised of a central elastic layer lined by 2 collagenous layers [7]. While the BM contains no lipids in early life, levels rise with age [29-31] and the BM can double in thickness by 90 years of age [32] as it calcifies and losses fluid permeability. Hence, thickening of BM is a salient feature of outer retinal aging.

**Figure 3:**
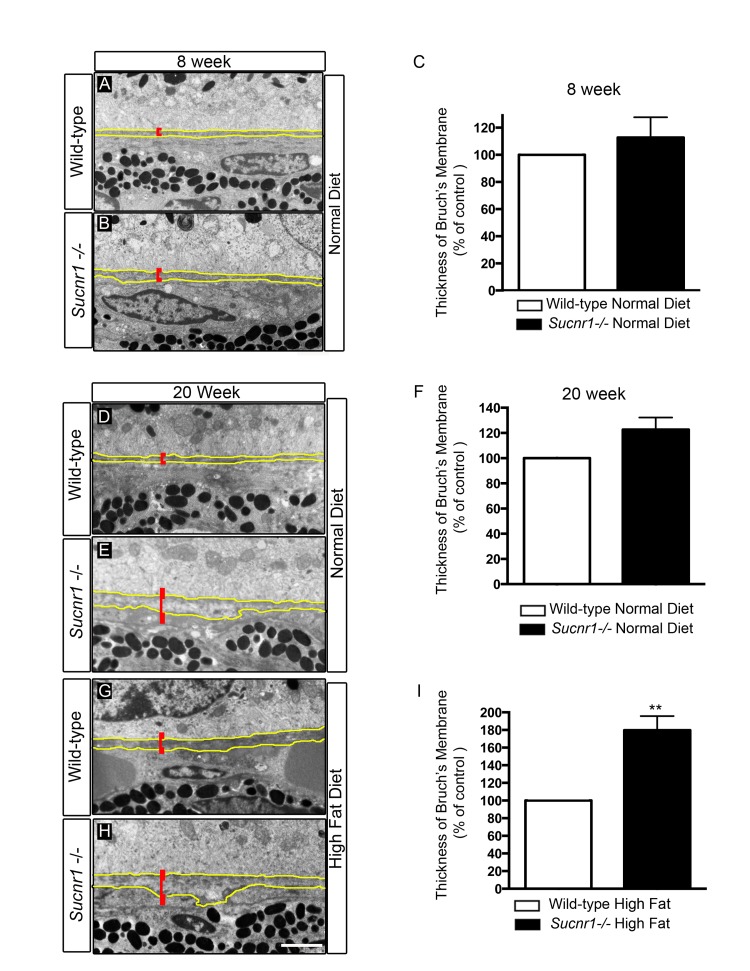
*Sucnr1−/−* mice develop Bruch's membrane thickening Transmission electron microscopy of BM (outlined in yellow) with annotated thickness bar (red). BM is systematically thicker in *Sucnr1−/−* mice at 8 weeks (**A-C**), and 20 weeks (**D-F**). High fat diet exacerbates the noted difference in BM thickness at 20 weeks (**G-I**). Data is compiled from 15 points of measure per retina in 4 distinct animals. **P<0,01. Scale bar: 1μm.

Thorough analysis of BM ultrastructure (15 points of measure per retina) revealed a pronounced thickening as determined by transmission electron microscopy in *Sucnr1−/−* mice when compared to wild-type controls. At 8 weeks of life, *Sucnr1−/−* mice showed only slightly thicker membranes than age matched controls (Figure 3A-C). At the relatively early age of 20 weeks, *Sucnr1- /-* mice had swollen BMs (~22% thicker than controls) (Figure 3D-F); an effect that was aggravated in animals receiving high fat diets (~80% thicker than controls; P=0.0021) (Figure 3G-I). Taken with the previous, our data suggest that deficiency in SUCNR1 leads to increased sub-retinal deposits and a paralleled thickening of BM.

### SUCNR1 Deficiency Leads to Compromised Microglial Motility and Provokes Subretinal Accumulation of Microglial Cells

**Figure 4:**
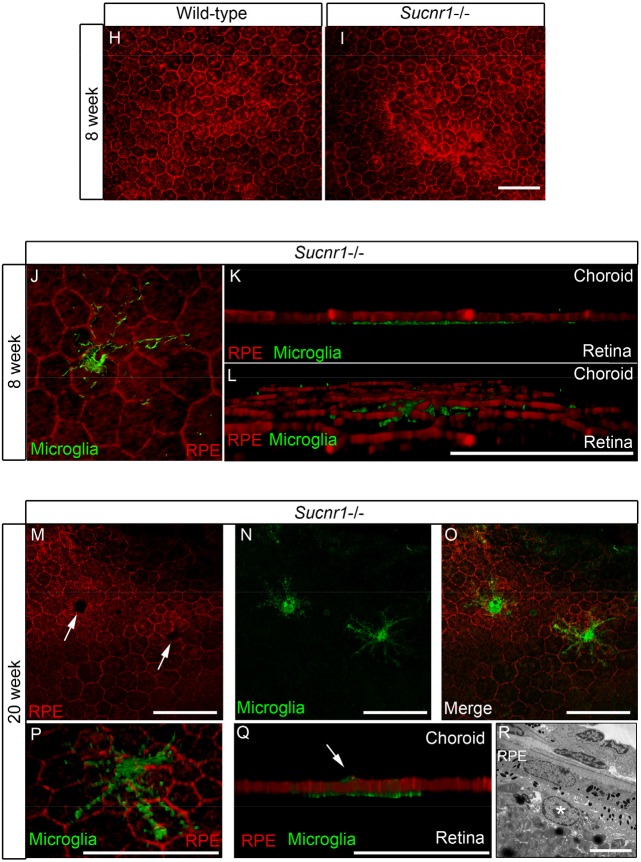
SUCNR1 deficient microglia show impaired migration and accumulate in the subretinal space (**A**) Migration assays performed in modified Boyden chambers demonstrate that macrophages from *Sucnr1−/−* mice have compromised chemotaxis towards oxLDL. The migratory potential of macrophages was not enhanced by treatment with succinate (100μM) (n=7). *P<0,05. (**B-C**) RPE flatmounts extracted at 8 weeks reveal a significant (**D**) accumulation of microglia in *Sucnr1−/−* mice when compared to wild-type controls. **P<0,01. Upon receiving high fat diets, the number of sub-retinal microglia did not vary (**G**) but were bloated (**E,F**). *P<0,05. Phalloidin stain of RPE flatmounts shows that RPE morphology is intact at 8 weeks in both knockout and controls (H,I), and that microglia (IBA1:green) remain on the retinal side of RPE (J-L). At 20 weeks, microglia (IBA1;green) puncture (**M-O**) and penetrate the RPE (P,Q). A portion of microglia remain on the retinal side of the RPE at 20 weeks as determined by transmission electron microscopy (R). Scale bar (**B,C,E,F**): 50μm,(**I**): 50μm, (**J-Q**) 20μm, (**R**): 5μm.

Microglia have been shown to partake in the pathogenesis of both dry and neovascular AMD[17, 18, 33]. Given the significant expression of SUCNR1 in microglia (Figure 1 and [21]) and evidence for the compromised migration of SUCNR1-deficient immune cells [21] we sought to determine if *Sucnr1−/−* macrophages became less mobile once in the sub-retinal space. In this regard, we investigated macrophage migration secondary to exposure to oxLDL (a component of extracellular drusen). Using a modified Boyden chamber, we observed that while wild-type macrophages readily crossed over towards an oxLDL-rich chamber, those derived from Sucnr1−/− mice did not migrate effectively (Figure 4A**;** P=0.0404). Interestingly, succinate (100μM) itself did not affect the overall mobility of macrophage in the assay employed and did not potentiate the effects of ox-LDL.

We next investigated the behavior of these immune cells within the outer retinas of *Sucnr1−/−* mice. Confocal microscopy on RPE flatmounts revealed that subretinal microglia accumulated ~6-fold more readily in *Sucnr1−/−* mice as early as at 8 weeks of life, (Figure 4B-D; P=0.0096). This increase persisted at 20 weeks of life (Figure 4E-G**;** P=0.0266). Subretinal microglia were ramified and formed dense clusters. From the 8^th^ week of life, mice were fed high fat diets (described above) to precipitate outer retinal degeneration. While high fat diets did not exacerbate accumulation of subretinal microglia (determined at 20 weeks of life), their morphology was altered and microglia appeared bloated. (Figure 4E-F).

Structural analysis of wild-type and *Sucnr1−/−* RPE at 4 weeks of life did not reveal any gross abnormalities with respect to layer integrity (Figure 4H-I). In addition, 3D rendering of the retinal-RPE interface confirmed microglial accumulation adjacent to photoreceptors (Figure 4J-L). In contrast, by 20 weeks, the RPE of *Sucnr1−/−* mice showed several zones local of compromise (Figure 4M**;** white arrows). These punctures in the RPE co-localized seamlessly with IBA1-positive microglia (Figure 4M-Q) underscoring the role of these cells in the progression of AMD-like outer retinal lesions. As expected, not all microglia breached the RPE as demonstrated by EM (Figure 4R**;** white asterix represents a vacuolated microglial cell in the sub-RPE). Consistent with the role of microglia in the progression of dry AMD and GA, our data demonstrate that Sucnr1−/− macrophages have compromised mobility and that *Sucnr1−/−* mice accumulate microglia in their subretinal space.

### Systemic Deficiency in SUCNR1 is Required for Subretinal Accumulation of Microglia

To determine the sequence of events leading to microglial accumulation in the sub retinal space, we proceeded to graft bone marrow from *Sucnr1−/−* mice into healthy wild-type controls and vice-versa. In doing so, we sought to determine if microglial cells amassed in Sucnr1−/− mice was secondary to the noted abnormal accumulation of oxLDL in the subretinal space (Figure 2) or a result of overall reduced mobility of SUCNR1 deficient microglia.

**Figure 5:**
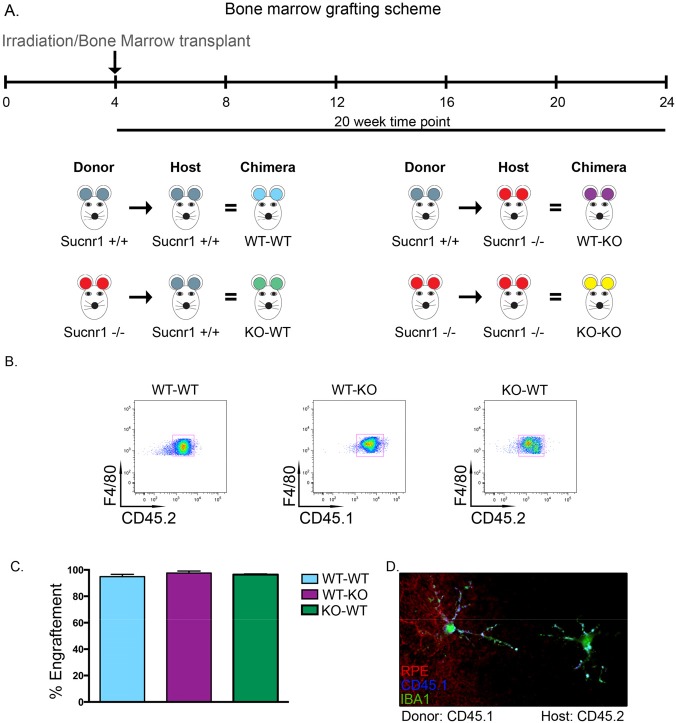
Systemic deficiency in SUCNR1 is required for subretinal accumulation of microglia (**A**) Depiction of bone marrow transplantation scheme. (**B**) Engraphtment efficiency is confirmed by FACS analysis of host blood and reveals that over 90% of hematopoietic cells in circulation are derived from donor cells as compiled in (**C**). (**D**) RPE flatmounts from a CD45.2 positive *Sucnr1−/−* host shows that donor microglia from CD45.1 mice have traversed and reside in host retinas. (**E**) Bone marrow transfer from wild-type into *Sucnr1−/−* hosts results in elevated levels of F480+CD11b+ cells in peripheral blood while transfer of *Sucnr1−/−* bone marrow into wild-type hosts does not affect numbers of blood-born macrophages. (n=3-4), *P<0,05. (**F**) Following bone marrow graphting, elevated levels of subretinal microglial were only noted when hosts were deficient in SUCNR1, (n=3) *P<0,05. (**G**) Graphic depiction of the sequence of events where *Sucnr1−/−* microglia accumulate in sub-retinal zones subsequent to drusen formation. Lesions of the outer retina then take place.

Hematopoietic precursors were transferred from donors at 4 weeks and animals were sacrificed 20 weeks post-transfer for RPE flat-mounting or FACS analysis. A schematic of the transfer scheme employed is depicted in Figure 5A. *Sucnr1−/−* mice are CD45.2 positive while wild-type mice were selected to be the inverse of their donors/recipients (either CD45.1 or CD45.2). Following bone marrow transfer, we first confirmed the engraftment efficacy by assessing the proportions of cell populations that were positive for F4/80 and CD45.1 or CD45.2. FACS analysis demonstrates that after transfer, over 90% of hematopoietic cells in the host's circulation are derived from donor cells, thus confirming the elevated efficiency of bone marrow engraftment in our experimental paradigm (Figure 5B&C). Interestingly, microglia of donor origin (CD45.1+) were found in RPE flat mounts of *Sucnr1−/−* host mice (CD45.2+), demonstrating that microglial cells which are found in the retina at the time of assessment (20 weeks post-graft), initially originated from the bone marrow (Figure 5D).

Interestingly, transfer of wild-type bone marrow into *Sucnr1−/−* hosts lead to a 2-fold increase in F480+CD11b+ cells in peripheral blood (P=0.0118). Conversely, upon transfer of *Sucnr1−/−* bone marrow into wild-type hosts, the numbers of peripheral blood macrophages remained at levels observed upon autologous wild-type to wild-type transfers and half that observed following autologous *Sucnr1−/−* transfer (Figure 5E; P=0.0427).

Similarly, analysis of RPE flatmounts subsequent to the transfer schemes outlined in Figure 5A, revealed that microglia accumulated uniquely when the host was deficient in SUCNR1. When wild-type marrow was transferred to *Sucnr1−/−* mice, the number of subretinal microglia was ~10-fold higher (P= 0.0217) while transfer of *Sucnr1−/−* to wild-type hosts did not increase microglial levels above wild-type controls (Figure 5F).

Together these results suggest that the subretinal accumulation of microglia is a function of systemic (and not microglial) deficiency in SUCNR1. Taken with the previous (Figure 4), our results point to a pivotal role for SUCNR1-deficient RPE in the progression of AMD-like outer-retinal degeneration. A graphic depiction of the sequence of events leading to dry AMD-like lesions of the subretinal space in *Sucnr1−/−* is shown in Figure 5G (see legend of Figure 5G for detailed explanation).

## DISCUSSION

Although AMD affects an estimated population base (in the USA) equal to cancer [1, 34] and double that suffering from Alzheimer's [35], the cellular and molecular mechanisms that precipitate outer retinal degeneration in GA and in late stage dry AMD remain incompletely understood. In this study, using a combination of human genetic analysis and mouse models, we provide evidence for the prospective involvement of SUCNR1 (GPR91) in preventing premature subretinal AMD-like lesions. Following analysis of 925 patients with GA and 1199 of their AMD-free peers, we report that human DNA sequence variants in *SUCNR1* (specifically rs13315275) are associated with atrophic AMD. In accordance, we demonstrate that *Sucnr1−/−* mice show signs of premature outer retinal degeneration with accumulation of subretinal oxLDL, thickening of BM and present abnormally elevated levels of subretinal microglial cells. The involvement of a metabolite receptor in the pathogenesis of AMD is consistent with a recent genome-wide association study that identified a significant association for SLC16A8, a monocarboxylic transporter for lactate [36].

Perturbation in the processing of lipids is associated with focal deposits in the BM and basal RPE deposits [37, 38]. Although a role for SUCNR1 in the regulation of white adipose tissue lipolysis has been suggested [22], in our experimental paradigm we found equivalent systemic levels of free fatty acids and glycerol in both *Sucnr1*-deficient and wild-type mice and hence did not observe compromised lipolysis. These findings are consistent with that of other metabolite sensing receptors [39] and thus point to regional retinal deficits in lipid processing secondary to SUCNR1 deficiency. Consistent with a role in preserving outer retinal integrity, SUCNR1 is expressed in RPE and microglia. Within the RPE, *Sucnr1* transcripts decrease as a function of age, suggesting an association with age-related RPE dysfunction. It has been proposed that there is an overlap in the molecular machinery involved in phagocytosis by RPE and by macrophages [40]. While SUCNR1 is expressed by both the RPE and microglia, it likely does not directly partake in the phagocytic process but rather regulates the propensity of RPE to uptake lipids. In line, RPE from *Sucnr1−/−* mice have significantly lower levels of the lipid scavenger receptor CD36 and accumulate oxLDL-rich deposits in their subretinal space. Absence of CD36 on RPE leads to BM thickening and drusen accumulation [11, 12]. Similarly, it has previously been observed in OXYS rats that RPE degeneration lead to choriocapillaris atrophy and later photoreceptor loss, suggesting a primary event in the RPE [41].

In agreement with the current understanding of myeloid cell dysfunction in AMD, Sucnr1−/− microglia show compromised mobility. Defects in microglial migration in *CXCR3−/−* mice have lead to cardinal features of AMD such as drusen formation, retinal degeneration and choroidal neovascularization[18]. Moreover, compromised microglial/macrophage recruitment from choroidal circulation in CCL-2 and CCR-2-deficient mice preclude the evacuation of debris from the BM [17]. However, although SUCNR1 is expressed on microglial cells and contributes to cellular migration in response to oxLDL, our data demonstrate that loss of SUCNR1 exclusively in microglia is insufficient to lead to sub-retinal microglial accumulation. In an attempt to elucidate the contribution of SUCNR1 deficient RPE versus microglia in disease progression, we proceeded to transfer bone marrow and hematopoietic precursors from *Sucnr1−/−* mice into wild-type controls. In doing so, we uncovered that the disease phenotype appeared only when hosts were deficient in SUCNR1. Correspondingly, transfer of *Sucnr1−/−* microglial into wild-type mice did not recapitulate the pathological phenotype, confirming that the trigger for disease progression lied within the retina proper (likely RPE cells given their expression of SUCNR1). Together with the previous, these findings point to SUCNR1 deficient RPE as the primary instigator of the pathological process and suggest that microglial accumulation is a consequence rather than primary cause of lesion.

While we provide human and animal evidence for the protective role of SUCNR1 against dry AMD-like lesions, it is important to specify that the overall retinal degeneration observed in *Sucnr1−/−* is relatively mild when compared to the sever retinal degeneration reported in mutants of CCL-2 and CCR-2[17] or CX3CR1[18]. It is nevertheless possible that deficiency in SUCNR1 predisposes and contributes to disease progression.

The control of metabolic pathways such as those of lipolysis, lipogenesis, fatty acid oxidation and synthesis requires a stringent orchestration. The discovery of cognate receptors for energy metabolites [20] offers a potential regulatory mechanism by which carbohydrates and lipids could play out hormone-like roles and regulate cellular processes[42]. Hence, investigation of carbohydrate metabolism in the context of degenerative ocular disease will likely contribute to the future mechanistic understanding of the disease process. Here we provide the first evidence for the protective role of a metabolite-sensing receptor, namely SUCNR1, against the progression of dry-form AMD. Understanding how deficiency in metabolite receptors leads to outer retinal lesion may offer future therapeutic avenues for this major cause of blindness and answer a clinically unmet need.

## MATERIALS AND METHODS

### Animals

All studies were performed according to the Association for Research in Vision and Ophthalmology (ARVO) Statement for the Use of Animals in Ophthalmic and Vision Research and were approved by the Animal Care Committee of the University of Montreal in agreement with the guidelines established by the Canadian Council on Animal Care. Mice were housed at local animal facilities under 12 hours light-12 hours dark cycles and fed ad libitum with a normal (ND) or a high fat (HF) (F3282, Bio-Serv, Frenchtown, NJ 08825). Total fatty acid content is: C18:2 Linoleic gm/kg 36.6, C18:3 Linolenic gm/kg 3.6, Total Saturated gm/kg 141, Total Monounsaturated gm/kg 162 and Total Polyunsaturated gm/kg 40.2.

### Generation of Suncr1−/− mice

Sucnr1−/− mice were generated by Deltagen by replacement of part of exon 2 (5'-GGCTACCTCTTCTGCAT-3') with a lacZ-neomycin cassette. Correctly targeted 129/OlaHsd embryonic stem cells were used for the generation of chimeric mice, which were crossed with C57BL/6 (called ‘wild-type’ here). F1 mice with germline transmission of the mutated gene were further backcrossed with wild-type mice for ten generations (in specific pathogen-free conditions at the Novartis Institutes for Biomedical Research, Vienna) before being inter- crossed to produce homozygous Sucnr1−/− mice. Wild-type, heterozygous and homozygous Sucnr1−/− mice were identified by ‘multiplex’ PCR with the following primers: GS (E1, forward), 5'-GAATTGGTTGGCAACAGAGGC TATC-3'; NEO (T, forward), 5'-GGGTGGGATTAGATAAATGCCTGCT CT-3'; and GS (E, T, reverse), 5'-TGCTGGTGTAGAG GTTGGTGTGAAG-3'. The wild-type Sucnr1 allele was identified by the presence of a 292-base pair DNA fragment, whereas the targeted Sucnr1 allele was identified by the presence of a 572-base pair DNA fragment. Sucnr1−/− mice were healthy and bred normally when maintained in specific pathogen–free conditions. All mouse experiments used mice 8-16 weeks of age. All in vivo studies were in accordance with the Austrian Law on Animal Experimentation and the Novartis Animal Welfare Policy. All procedures were approved by the local government and the animal care and user committee of the Novartis Institutes for Biomedical Research, Vienna.

### Semi-quantitative and Real-time PCR analysis

RNA was isolated using Trizol (Invitrogen) and digested with DNase I to prevent amplification of genomic DNA. Reversed transcription was performed using M-MLV reverse transcriptase and gene expression analyzed using SybrGreen in an ABI Biosystems Real-Time PCR machine. β-actin was used as a reference gene.

### Immunohistochemistry

To localize protein expression, eyes were enucleated from mice and fixed in 4% paraformaldehyde at room temperature for 4h and incubated in 30% sucrose overnight and then frozen in OCT compound. We then embedded the whole eye in optimal cutting temperature compound at −20°C and performed 12um serial sections. We carried out immunohistochemistry experiments and visualized the sections with an epifluorescent microscope (Zeiss AxioImager) or confocal microscope (Olympus confocal FV1000) and 3D rendering performed using Volocity software. Antibodies were: SUCNR1 (Santa Cruz, #sc-50466), RPE65 (ABCAM, #ab13826), Ox-LDL (Millipore, #AB3230), Iba1 (Wako Chemical Industries ltd., #019-19741), Rhodamine-Phalloidin (Biotium, inc., #00027), CD45.1 (BioLegend, #110701), DAPI (Invitrogen, #D1306)rat monoclonal anti-CD11b (Serotec), rat anti-F4/80 (BD Biosciences – Pharmingen).

### Histology and electron microscopy

For histology and electron microscopy, eyes were fixed in a solution of sodium cacodylate 0,1M with glutaraldehyde 3,5%,dehydrated, and mounted in historesin. The samples were included in epoxy resin and oriented. Semithin sections (1 μm) were sliced with an ultramicrotome Reichert Ultracut E (Leica), stained by toluidine blue, and examined with a light microscope to measure the photoreceptor layer thickness. Multiple measurements of the photoreceptor cell were used to calculate the relative retinal thicknesses at sites 100-1,000 microns from the ONH. Ultrathin sections (80 nm) were stained for contrast with uranyl acetate and lead citrate and were observed in a JEOL 100 CX II electron microscope (JEOL) with 80 kV.

### RPE isolation

RPE cells were isolated from mouse pups at post-natal days 9 to 14 and were age-matched between wild-type and knockout mice. Eyes were enucleated and incubated overnight in DMEM + 10% FBS + 0,2% fungizone in dark conditions at room temperature. Eye balls were cleaned of conjunctive tissue before digestion with collagenase IV (2 mg/mL) and trypsin (2 mg/mL) for 30 minutes at 37°C. Corneas, lens and retina were removed, leaving only RPE layer and choroid. The RPE layer was carefully peeled from sclera, and the remaining vessels were removed. RPE sheets were collected and disrupted by repeated pipetting. The resulting cell suspension was plated and cultured for 1 week at 37°C prior to use in experiments.

### Isolation of primary microglia

Brains from P14 mouse pups were homogenized in ice-cold L15 medium (Gibco) and incubated them for 15min in 0,05% trypsin-EDTA (Gibco) at 37°C. The reaction was stopped by addition of equal volumes of fetal bovine serum. We added 75U/mL of DNase I (Sigma) before filtering through a 70μm cell strainer. After 9 days of incubation in DMEM media (Invitrogen) supplemented with fetal bovine serum 10% and penicillin/streptomycin, microglia were detached from the plates by gentle shaking (150rpm for 2hrs at 37°C). Purity of preparations was confirmed by FACS analysis.

### Glycerol and Free fatty acid quantification

Mice were fasted for 12 hours during light cycle, followed by intra-peritoneal administration of 3 mg/g glucose for 30 minutes at the beginning of the dark cycle.

Blood was collected in EDTA treated tube, centrifuged at 1,000 × g for 10 minutes at 4°C, and top yellow plasma layer was carefully isolated. Samples were run through enzymatic reactions according to instructions provided with the Glycerol Colorimetric Assay Kit (Caymen Chemical Company). Absorbance was measured at 550 nm with a SpectraMax spectro-photometer. Samples were also analyzed for free fatty acid quantification using a Free Fatty Acid Assay Kit (Caymen Chemical Company). Fluorescence was measured (ex. 530, em. 585) with a CytoFluor 4000 fluorometer.

### Macrophage migration assay

Macrophages were harvested from *Sucnr1−/−* or wild-type mice following peritoneal washes. Migration was assessed using a modified Boyden chamber method. Briefly, 5×10^5^ macrophages were placed on top of an 8μm transwell chamber and incubated overnight with the chemotactic agent in the bottom chamber. Migrated cells were coloured using Hema-Stain and counted.

### Bone marrow chimera

For the generation of chimeric mice, bone marrow cells were obtained by flushing both tibias, femurs and iliac crests of wt C57/B6 CD45.1 or CD45.2 or SUCNR1^−/−^ donor mice. 4 week old C57/B6

CD45.2 or SUCNR1^−/−^ recipient mice were irradiated with 8 Gy from a 6MV linear particle accelerator (Elekta) and injected intravenously with 1×10^7^ bone marrow cells. Chimeric mice were sacrificed 20 weeks later.

### Flow cytometry

Blood leukocytes populations were analyzed using a BD LSRII flow cytometer and postanalysis was done using the FlowJo 7.6 software. Compensation was done using single-stained cells. Leukocyte were stained with antibodies (all from BioLegend) against CD11b-FITC (clone M1/70), CD11c-PE (clone N418), Gr-1-PerCP (clone RB6-8C5) and F4-80-Pacific Blue (clone CI:A3-1). Cells were first gated for CD11b+/CD11c- population. Then, Gr-1 low or intermediate and F4-80+ cells were counted as macrophages. For calculation of bone-marrow transfer efficiencies, cells were also stained with CD45.1-APC (clone A20) or CD45.2-PE-Cy7 (clone 104) antibodies.

### Statistical analyses

Data are presented as mean ± s.e.m. We used Student's T-test to compare the different groups; a P < 0.05 was considered statistically different.

### Human genetic analysis

*Data used for genetic analyses* in this report were obtained from the NEI Age-Related Eye Disease Database (NEI-AREDS) Database and the NEI Study of Age-Related Macular Degeneration (NEI-AMD) Database at the U.S. National Center for Biotechnology Information (NCBI) database of Genotypes and Phenotypes (dbGaP). Details are presented in the Supplementary Information.

## SUPPLEMENTARY INFORMATION


