# Supplementary material for: Deficiency in the metabolite receptor SUCNR1 (GPR91) leads to outer retinal lesions
Source: Aging (Albany NY). 2013 Jun 17;5(6):427–44. doi: 10.18632/aging.100563 (PMC3832265; doi:10.18632/aging.100563)
Supplement: Supplementary file 1 [file aging-05-427-s001.pdf]

drusen and possible involvement of anti-retinal autoimmunity in two different forms of macular degeneration in cynomolgus monkey (Macaca fascicularis). FASEB J. 2005; 19:1683-1685.
17. Ambati J, Anand A, Fernandez S, Sakurai E, Lynn BC, Kuziel WA, Rollins BJ and Ambati BK. An animal model of age-related macular degeneration in senescent Ccl-2- or Ccr-2-deficient mice. Nat Med. 2003; 9:1390-1397.
18. Combadiere C, Feumi C, Raoul W, Keller N, Rodero M, Pezard A, Lavalette S, Houssier M, Jonet L, Picard E, Debre P, Sirinyan M, Deterre P, et al. CX3CR1-dependent subretinal microglia cell accumulation is associated with cardinal features of age-related macular degeneration. J Clin Invest. 2007; 117:2920-2928.
19. Lavalette S, Raoul W, Houssier M, Camelo S, Levy O, Calippe B, Jonet L, Behar-Cohen F, Chemtob S, Guillonneau X, Combadiere C and Sennlaub F. Interleukin-1beta inhibition prevents choroidal neovascularization and does not exacerbate photoreceptor degeneration. Am J Pathol. 2011; 178:2416-2423.
20. He W, Miao FJ, Lin DC, Schwandner RT, Wang Z, Gao J, Chen JL, Tian H and Ling L. Citric acid cycle intermediates as ligands for orphan G-protein-coupled receptors. Nature. 2004; 429:188-193.
21. Rubic T, Lametschwandtner G, Jost S, Hinteregger S, Kund J, Carballido-Perrig N, Schwarzler C, Junt T, Voshol H, Meingassner JG, Mao X, Werner G, Rot A, et al. Triggering the succinate receptor GPR91 on dendritic cells enhances immunity. Nat Immunol. 2008; 9:1261-1269.
22. Regard JB, Sato IT and Coughlin SR. Anatomical profiling of G protein-coupled receptor expression. Cell. 2008; 135:561-571.
23. Sapieha P, Sirinyan M, Hamel D, Zaniolo K, Joyal J-S, Cho J-H, Honoré J-C, Kermorvant-Duchemin E, Varma DR, Tremblay S, Leduc M, Rihakova L, Hardy P, et al. The succinate receptor GPR91 in neurons has a major role in retinal angiogenesis. Nature Medicine. 2008; 14:1067-1076.
24. Priya RR, Chew EY and Swaroop A. Genetic studies of age-related macular degeneration: lessons, challenges, and opportunities for disease management. Ophthalmology. 2012; 119:2526-2536.
25. Gnana-Prakasam JP, Ananth S, Prasad PD, Zhang M, Atherton SS, Martin PM, Smith SB and Ganapathy V. Expression and iron-dependent regulation of succinate receptor GPR91 in retinal pigment epithelium. Invest Ophthalmol Vis Sci. 2011; 52:3751-3758.
26. Wolf G. Lipofuscin and macular degeneration. Nutr Rev. 2003; 61:342-346.
27. Malek G, Johnson LV, Mace BE, Saloupis P, Schmechel DE, Rickman DW, Toth CA, Sullivan PM and Bowes Rickman C. Apolipoprotein E allele-dependent pathogenesis: a model for age-related retinal degeneration. Proc Natl Acad Sci U S A. 2005; 102:11900-11905.
28. Mattapallil MJ, Wawrousek EF, Chan CC, Zhao H, Roychoudhury J, Ferguson TA and Caspi RR. The Rd8 mutation of the Crb1 gene is present in vendor lines of C57BL/6N mice and embryonic stem cells, and confounds ocular induced mutant phenotypes. Invest Ophthalmol Vis Sci. 2012; 53:2921-2927.
29. Bird AC and Marshall J. Retinal pigment epithelial detachments in the elderly. Trans Ophthalmol Soc U K. 1986; 105:674-682.
30. Holz FG, Sheraidah G, Pauleikhoff D and Bird AC. Analysis of lipid deposits extracted from human macular and peripheral Bruch's membrane. Arch Ophthalmol. 1994; 112:402-406.
31. Pauleikhoff D, Harper CA, Marshall J and Bird AC. Aging changes in Bruch's membrane. A histochemical and morphologic study. Ophthalmology. 1990; 97:171-178.
32. Ramrattan RS, van der Schaft TL, Mooy CM, de Bruijn WC, Mulder PG and de Jong PT. Morphometric analysis of Bruch's membrane, the choriocapillaris, and the choroid in aging. Invest Ophthalmol Vis Sci. 1994; 35:2857-2864.
33. Tuo J, Bojanowski CM, Zhou M, Shen D, Ross RJ, Rosenberg KI, Cameron DJ, Yin C, Kowalak JA, Zhuang Z, Zhang K and Chan CC. Murine ccl2/cx3cr1 deficiency results in retinal lesions mimicking human age-related macular degeneration. Invest Ophthalmol Vis Sci. 2007; 48:3827-3836.
34. Hayat MJ, Howlader N, Reichman ME and Edwards BK. Cancer statistics, trends, and multiple primary cancer analyses from the Surveillance, Epidemiology, and End Results (SEER) Program. Oncologist. 2007; 12:20-37.
35. Brookmeyer R, Johnson E, Ziegler-Graham K and Arrighi HM. Forecasting the global burden of Alzheimer's disease. Alzheimers Dement. 2007; 3:186-191.
36. Consortium DG, Fritsche LG, Chen W, Schu M, Yaspan BL, Yu Y, Thorleifsson G, Zack DJ, Arakawa S, Cipriani V, Ripke S, Igo RP, Jr., Buitendijk GH, et al. Seven new loci associated with age-related macular degeneration. Nat Genet. 2013; 45:433-439.
37. Curcio CA, Johnson M, Rudolf M and Huang JD. The oil spill in ageing Bruch membrane. Br J Ophthalmol. 2011; 95:1638-1645.
38. Kishan AU, Modjtahedi BS, Martins EN, Modjtahedi SP and Morse LS. Lipids and age-related macular degeneration. Surv Ophthalmol. 2011; 56:195-213.
39. Ahmed K, Tunaru S, Tang C, Muller M, Gille A, Sassmann A, Hanson J and Offermanns S. An autocrine lactate loop mediates insulin-dependent inhibition of lipolysis through GPR81. Cell Metab. 2010; 11:311-319.
40. Kevany BM and Palczewski K. Phagocytosis of retinal rod and cone photoreceptors. Physiology (Bethesda). 2010; 25:8-15.
41. Markovets AM, Saprunova VB, Zhdankina AA, Fursova A, Bakeeva LE and Kolosova NG. Alterations of retinal pigment epithelium cause AMD-like retinopathy in senescence-accelerated OXYS rats. Aging (Albany NY). 2011; 3:44-54.
42. Blad CC, Tang C and Offermanns S. G protein-coupled receptors for energy metabolites as new therapeutic targets. Nat Rev Drug Discov. 2012; 11:603-619.

SUPPLEMENTARY INFORMATION

Human genetic analysis

Details on NEI-AREDS exist at:
http://www.ncbi.nlm.nih.gov/projects/gap/cgibin/study.cgi?study_id=phs000001.v2.p1.
NEI-AREDS was a collaborative of scientists and clinicians at the AREDS Project Office NEI Clinical Trials Branch, the AREDS Coordinating Center, the AREDS Photographic Reading Center, the NEI Office of the Director, and the AREDS clinical sites.

Details on NEI-AMD exist at:
www.ncbi.nlm.nih.gov/projects/gap/cgibin/study.cgi?study_id=phs000182.v2.p1.

NEI-AMD is a collaborative of researchers from the University of Michigan, Mayo Clinic, University of Pennsylvania, and the AREDS group including National Eye Institute intramural investigators. Institutional review boards at each NEI-AMD study site reviewed and approved the study protocols. Each participant provided written informed consent in accordance with the *Declaration of Helsinki*.

Subjects and Study Design

NEI-AREDS Cohort. Details on AREDS and the demographics NEI-AREDS participants involved in the NEI-AREDS genome-wide association study design exist at the link cited above. In brief, AREDS was a 12 year multi-center natural history study (with a 5-year phase III clinical trial) on 4757 elderly U.S. residents designed to assess the clinical course of, and risk factors for, the development and progression of AMD by collecting data on possible risk factors, measuring changes in visual acuity, photographically documenting changes in macula status, and assessing self-reported visual function. Eleven retinal specialty clinics enrolled participants aged 55 to 80 years from November 1992 through January 1998, and followed them until April 2001. A natural history study extending to December 2005 was implemented in April 2001. Our analytic sample contained 391 people with GA or GA+NV AMD and 189 of their AMD-free peers.

NEI-AMD Cohort. Details on the NEI-AMD genome-wide association (GWA) study exist at the link in the first paragraph of this section. In brief, three independent cohorts from the University of Michigan in Ann Arbor, the University of Pennsylvania in Philadelphia, and the Mayo Clinic in Rochester, Minnesota contributed data to this genome-wide association study. Our analytic sample contained respectively 329, 110, and 95 people with GA or GA+NV AMD from the University of Michigan, University of Pennsylvania, and The Mayo Clinic. There were 508, 194, and 308 AMD-free people, aged $\geq$ 65 years from these respective sites.

Outcome Ascertainment

We restricted our AMD-free comparison cohort to people aged $\geq$ 65 years. The likelihood of having AMD increases 2-to-6 fold after age 75 and it was therefore essential to select our oldest AMD-free participants to reduce the chances of including false negatives in analyses (that would otherwise result from non-random misclassification in the youngest members of the control group).

AREDS Cohort. AREDS Report 1 contains information on outcome ascertainment in the NEI-AREDS cohort. Specifics on the classification of GA exist at https://web.emmes.com/study/areds/mopfiles/chp15_mop.pdf

Geographic atrophy was classified as one or more sharply defined, usually more or less circular patches of partial or complete depigmentation of the retinal pigment epithelium (RPE), typically with exposure of underlying large choroidal blood vessels -- either at baseline or during the course of the study. To be classified as geographic atrophy in AREDS, a patch had to be at least as large in area as Circle I-1 (see link directly above). In general, at least two of the characteristics mentioned (sharp edges, more or less circular shape, and visibility of underlying choroidal vessels) were required for a patch to be classified as geographic atrophy -- if much of the RPE appeared to be preserved and large choroidal vessels were not visible, a roundish patch of RPE depigmentation with sharp edges may still have been classified as geographic atrophy. "Edge sharpness" was defined in either of two ways: (1) when the depigmentation within the patch was subtle, a "sharp" edge needed to be abrupt and smooth, like one made with a cookie-cutter, but (2) when contrast between depigmentation within a patch and the normal pigmentation around it was substantial, the edge of the patch may still have been considered "sharp", even if the transition occurred gradually or irregularly over a zone 125 to 250 μm in width. Increased visibility of large choroidal vessels was the single most important criterion and, when present, it was not necessary for all the edges of the patch to be classified as sharp.

Our NEI-AREDS controls have three distinguishing characteristics:

- Phenotype was determined annually over a 12-year period (AREDS) or across an 8-year period with a standardized protocol by multiple professional graders who were masked to phenotypic information from previous years. Adjudication with a standardized protocol occurred when discrepancies emerged.
- The criteria for AMD-free classification (< 5 drusen of $\leq$ 63 μm in both eyes for the entire follow-up period) is stringent relative to those applied in previous association studies for AMD.
- The age of the AREDS AMD-free group is in the range in which AMD prevalence increases ~3 times (from ~4% in those age 74-to-79 to ~12% in those $\geq$ 80-year-of-age) in population-based studies.

NEI-AMD Cohorts. Experienced graders (ophthalmologists) classified outcomes according to AMD diagnosis in the worse eye. Our AMD-free comparison group was composed of people $\geq$ 65-years-of-age who had no large

or intermediate drusen in either eye; these participants received examinations and gradings by the study ophthalmologists. If small drusen or pigment changes were present in the AMD-free group, they were neither bilateral nor extensive (≤ 5). Our geographic atrophy group had GA or GA + NV AMD in at least one eye; if the participant had unilateral advanced AMD, then this person was required to have drusen or pigment changes in the fellow eye. No participant exhibited history or evidence of: 1) retinal insult rendering the fundus ungradable; 2) severe macular disease or vision loss onset prior to 40-years-of-age; or 3) diagnosis of juvenile macular or retinal degeneration, macular damage resulting from ocular trauma, retinal detachment, high myopia, chorioretinal infection, or inflammatory disease, or choroidal dystrophy.

Genotyping

All NEI-AREDS and NEI-AMD specimens were genotyped with DNA microarrays at the Johns Hopkins University Center for Inherited Disease Research (CIDR, Baltimore, MD, USA).

NEI-AREDS Cohort. The NEI-AREDS cohort was genotyped with AFFYMETRIX Mapping50K_Hind240 (SNP batch IDs at http://www.ncbi.nlm.nih.gov/SNP/-snp_viewBatch.cgi?sbid=33750),

AFFYMETRIX Mapping50K_Xba240 (SNP batch IDs at http://www.ncbi.nlm.nih.gov/SNP/snp_viewBatch.cgi?sbid=33751), and

ILLUMINA ILMN_Human-1 (SNP batch IDs at http://www.ncbi.nlm.nih.gov/SNP/snp_viewBatch.cgi?sbid=33668).

NEI AMD Cohort. The NEI-AMD cohort was genotyped using ILLUMINA HumanCNV370v1 (SNP batch IDs at www.ncbi.nlm.nih.gov/SNP/snp_view-Batch.cgi?sbid=1047132) using the Illumina Infinium II assay protocol. The Illumina BeadStudio Genotyping module (version 3.2.32) was used to with the combined intensity of 99% of the samples to assign allele cluster definitions. The threshold for genotype calls was a gencall score ≥ 0.25. Reproducibility of blind duplicate samples was 99.992%.

Statistical Analysis of Succinate Receptor 1 (SUCNR1, GPR91)

We used Plink (version 1.07, pngu.mgh.harvard.edu.purcell/plink/) and SAS (version 9.1, Cary, NC) software for analysis.
The analytic plan was implemented as follows:

1. All sequence variants analyzed for the current study passed process quality and analytic filters for missingness (< 5%), minor allele frequency (> 1%) and Hardy-Weinberg equilibrium (HWE $P \leq 1 \times 10^{-6}$ in the AMD-free group).
2. We used the positional coordinates of SUCNR1 (± 1000 base pairs) to filter all high quality resident SNPs from genome-wide microarray data.
3. We examined the allelic distributions of these SNPs in people with GA (relative to the AMD-free comparison group) with age-, sex-, and smoking-adjusted logistic regression analyses using additive, dominant (grouping minor allele homozygotes with heterozygotes), and recessive (grouping major allele homozygotes with heterozygotes) models in each cohort.
4. Combined effects were estimated with age-, sex-, and smoking-adjusted meta-regression using Plink. All estimates were based on combining findings from the same model (e.g. findings from the additive model in one cohort were never combined with findings from different models in other cohorts). Sample heterogeneity was assessed with Cochrane's Q and random effects models were applied when indicated.
5. We computed exact *P*-values on AAMD-associated variants using a max(T) permutation procedure set to 10000 iterations.
